# Mast Cells at the Crossroad of Gut-Derived Signals Through Aryl Hydrocarbon Receptor Activation: A Microbial–Immune Dialogue in Liver Inflammation with Therapeutic Perspectives

**DOI:** 10.3390/cells15050449

**Published:** 2026-03-03

**Authors:** Francesco Vasuri, Barbara Frossi, Luca Saragoni, Giorgia Gri

**Affiliations:** 1 Department of Medical and Surgical Sciences (DIMEC), University of Bologna, 40126 Bologna, Italy; francesco.vasuri2@unibo.it (F.V.); luca.saragoni7@unibo.it (L.S.); 2 Pathology Unit, Santa Maria delle Croci Hospital, 48121 Ravenna, Italy; 3 Immunology Section, Department of Medicine, University of Udine, 33100 Udine, Italy; barbara.frossi@uniud.it

**Keywords:** aryl hydrocarbon receptor, chronic hepatitis, gut–liver axis, inflammatory microenvironment, mast cells

## Abstract

**Highlights:**

**What are the main findings?**

- Aryl hydrocarbon receptor (AhR) signaling may act as a mechanistic bridge linking gut-derived tryptophan metabolites to mast cell (MC) activation, potentially shaping hepatic inflammation and fibrogenesis.
- MCs may represent an underexplored node in the gut–liver axis, where the existing evidence on the roles of AhR in liver disease and MCs in liver pathology has rarely been integrated into a unified framework.

**What are the implications of the main findings?**

- Modulating microbial or dietary sources of endogenous AhR ligands, alongside selective AhR-targeting strategies, could help recalibrate MC-driven hepatic inflammation.
- Pending the validation of MC-specific AhR programs and disease-context outputs, AhR modulation may evolve into predictive or precision approaches for autoimmune and cholestatic liver diseases.

**Abstract:**

Mast cells (MCs) are multifunctional innate immune cells that regulate inflammation, tissue repair, and immune responses, and they are increasingly recognized as contributors to chronic liver disease. In parallel, the aryl hydrocarbon receptor (AhR) has emerged as a key environmental sensor activated by gut-derived tryptophan metabolites such as kynurenine and microbial indoles. The current literature separately describes the role of AhR in MC signaling, as well as the contributions of MCs to liver pathology and the disrupted gut–liver axis, which drives immune dysfunction in chronic liver disease. However, these aspects have been rarely considered together. This review aims to bridge these fragmented areas, providing an integrated framework where AhR-driven MC responses are examined within the gut–liver axis along with their impacts on liver inflammation and fibrosis. We discuss how this microbial–immune dialogue shapes autoimmune and cholestatic liver diseases, including autoimmune hepatitis, primary sclerosing cholangitis, and primary biliary cholangitis. Finally, we highlight translational perspectives, from microbiota modulation to AhR-targeting approaches, as potential strategies to control MC-driven hepatic inflammation. By integrating these currently separate concepts, this review offers a novel perspective on the role of MCs as important mediators at the interface of gut-derived signals and liver pathology via AhR signaling, while highlighting innovative therapeutic avenues through the modulation of the microbiota, targeting of AhR, and regulation of MC responses.

## 1. Introduction

Liver inflammation is the key driver of chronic liver diseases, with both immune and metabolic pathways contributing to its onset and progression. Among the signaling mechanisms involved, the aryl hydrocarbon receptor (AhR) emerged as a critical regulator of inflammation. As a ligand-activated transcription factor responsive to environmental and endogenous molecules, including tryptophan metabolites such as kynurenine, AhR stands at the interface of metabolism, immunity, and inflammation. Kynurenine is a product of tryptophan metabolism by the enzymes indoleamine 2,3-dioxygenase (IDO) and tryptophan 2,3-dioxygenase (TDO), and it can activate AhR, leading to the suppression of T-cell activity and inhibition of inflammatory signaling pathways (such as NF-kB) [1,2]. AhR signaling also modulates the innate immune system, influencing the activation of monocytes, neutrophils, and mast cells (MCs). Under physiological conditions, the AhR–kynurenine system contributes to the regulation and resolution of liver inflammation by maintaining immune tolerance and preventing excessive tissue damage. However, the dysregulated or sustained activation of this pathway may exert divergent effects depending on the ligand concentration, cellular context, and disease stage [3].

This review focuses on the role of the AhR–kynurenine axis in regulating liver inflammation, its interactions with innate immune cells and MCs, and its emerging connection with the gut microbiota. In particular, we will explore this latter pathway, as to date, no review has addressed the role of MCs in mediating microbiota-derived AhR signaling in liver pathophysiology.

## 2. Review Method

A narrative review methodology was employed. A literature search in the English language of PubMed/Medline and Scopus was conducted (inception through 31 December 2025), integrating terminology for AhR, chronic liver disease, gut–liver axis, hepatitis, inflammatory microenvironment, and mast cells. Equivalent logic (including MeSH terms and free-text entries) was used in both databases, along with a search in the Consensus AI-powered search engine “https://consensus.app” (accessed on 20 December 2025). The final selection prioritized peer-reviewed, non-retracted works to offer a comprehensive, pragmatic, and pluralistic overview of the topic.

## 3. AhR Structure, Function, and Environmental Sensing: A Gateway for Mast Cell Activation

AhR is a ligand-activated transcription factor belonging to the basic helix-loop-helix/Per-ARNT-Sim (bHLH-PAS) family. It acts as a versatile environmental sensor, integrating environmental and cellular signals to regulate gene expression. Structurally, AhR typically resides in the cytoplasm within a complex of chaperone proteins that stabilizes the receptor and maintains its ligand-responsive state. The PAS-B domain of AhR is crucial for ligand binding, facilitating interactions with a wide array of ligands. These ligands originate from diverse sources, such as environmental pollutants, dietary components, and even endogenous metabolites [4,5,6,7,8]. Upon ligand binding, AhR undergoes to conformational changes, dissociates from its chaperones, and translocates to the nucleus. Here, it dimerizes with the aryl hydrocarbon receptor nuclear translocator (ARNT). The resulting AhR-ARNT heterodimer forms an intertwined, asymmetric architecture that enables specific recognition and binding to DNA response elements, such as the dioxin response element (DRE), thereby initiating gene transcription [4,5,6,7,8].

The primary function of this structurally dynamic receptor involves the regulation of genes crucial for xenobiotic metabolism, including those encoding drug-metabolizing enzymes and transporters. This process aids in the detoxification and elimination of harmful compounds. Beyond this canonical role, AhR also modulates several physiological processes, such as immune regulation, cellular differentiation, metabolism, and organ development. The functional outcome of AhR activation is influenced by the nature and strength of its ligands, the specific cellular context, and the presence of coregulators and other transcription factors. AhR can participate in both canonical signaling (via ARNT and DRE binding) and non-canonical pathways, interacting with other signaling molecules to shape gene expression in a ligand-, cell-, and tissue-specific manner. This multifaceted functionality positions AhR as a key regulator of both health and disease, with significant implications for immunity, inflammation, and toxicological responses [8,9,10,11,12,13].

Formerly, AhR had been recognized as a receptor for xenobiotics and environmental pollutants such as dioxin (e.g., TCDD). Nevertheless, in recent years, a significant (and increasing) number of additional non-toxic molecules was identified as AhR ligands. These include dietary metabolites and microbial-derived compounds, which are endogenously generated in the gut by commensal microbiota [14]. Table 1 summarizes the major AhR ligands, their site of origin, route of delivery to the liver, and immunomodulatory effects [15,16,17,18,19,20,21,22,23,24,25,26,27,28,29].

Among these endogenous ligands, kynurenine, an indole metabolite of tryptophan produced by enzymes like indoleamine 2,3-dioxygenase (IDO) and tryptophan 2,3-dioxygenase (TDO), strongly activates AhR responses. Approximately 95% of the tryptophan not used for protein synthesis is degraded via the kynurenine pathway, and liver is the main organ involved in this mechanism. AhR activation by kynurenine notably modulates immune responses in both innate and adaptive compartments. In the liver, this can lead to either the suppression or amplification of inflammation, depending on the context. Kynurenine–AhR signaling has specifically been shown to support immunosuppression and tolerance, particularly in settings of chronic inflammation and cancer [30,31,32,33].

All ligands listed in Table 1 are established AhR agonists, but the mechanistic evidence for direct MC modulation is heterogeneous across ligand classes. Endogenous and dietary ligands (e.g., kynurenine, FICZ, and resveratrol) exhibit demonstrated AhR-dependent effects on MC activation and mediator release in experimental models, a topic that is addressed in Section 5.2, whereas prototypical environmental toxicants such as TCDD and benzo[a]pyrene lack robust, cell-intrinsic evidence for direct modulation of MC function.

## 4. Physiological and Pathological Roles of AhR in the Liver: Providing the Context for Mast Cell Activity

To contextualize how MCs operate within the liver environment, this section summarizes core AhR functions in hepatic parenchymal and non-parenchymal cells, highlighting how these processes shape the metabolic and inflammatory milieu encountered by periportal and peribiliary MCs and thereby influence the MC response to gut-derived signals.

### 4.1. Hepatic AhR Expression and Core Physiological Roles

As a sensor of environmental and metabolic cues, AhR is highly expressed in barrier organs such as the gut, skin, and lung, as well as in organs that are steadily exposed to potentially harmful endogenous and exogenous compounds, including the liver. In the hepatic compartment, AhR is constitutively expressed in multiple parenchymal and non-parenchymal cell types, where it plays a pivotal role in normal liver development and in the maintenance of hepatic homeostasis. Proteomic profiling confirms appreciable AhR expression not only in hepatocytes but also in liver sinusoidal endothelial cells (LSECs), hepatic stellate cells (HSCs), and, to a lesser extent, cholangiocytes, underscoring the broad cellular distribution of this receptor within the liver [33]. A comprehensive overview of AhR-expressing hepatic cell populations is provided in Table 2.

In hepatocytes, the primary function of AhR involves regulating the expression of detoxification enzymes, including CYP1A1, which are essential for xenobiotic clearance and cellular protection [34,35,36]. Beyond its canonical role in detoxification, AhR contributes to fundamental hepatic processes, such as mitochondrial homeostasis, redox balance, and metabolic regulation [34,35,36].

The importance of AhR for liver physiology is strongly supported by phenotypic abnormalities observed in AhR-knockout mouse models, which display reduced liver and hepatocyte sizes, an immature sinusoidal architecture, incomplete closure of the neonatal ductus venosus, and marked portosystemic shunting [37,38]. These defects highlight a critical role for AhR in coordinating hepatic growth, vascular maturation, and structural integrity. Consistent with these observations, proteomic analyses of whole-body AhR-knockout mice identify AhR as a principal regulator of lipid, organic acid, and xenobiotic metabolism, as well as bioenergetic and endocrine pathways [39].

Because periportal and peribiliary MCs are embedded within AhR-responsive hepatic niches, the receptor’s activity in hepatocytes, LSECs, cholangiocytes, and HSCs critically shapes the signals—metabolic intermediates, cytokines, and damage-associated cues—that MCs may integrate during liver inflammation.

### 4.2. Metabolic Regulation

AhR exerts a significant, multifaceted influence on hepatic metabolism, playing key roles in lipid, fatty acid, and mitochondrial homeostasis. Specifically, AhR represses the expression of genes integral to fatty acid synthesis. By attenuating the overall fatty acid synthesis and the subsequent secretion in hepatocytes, AhR contributes to maintaining lipid homeostasis and preventing metabolic diseases, particularly steatosis [13,40,41]. This repressive role highlights its potential as a therapeutic target for metabolic liver diseases. Furthermore, AhR supports mitochondrial homeostasis by regulating mitophagy, the selective removal of damaged mitochondria, which is essential for ensuring robust hepatic energy balance [34]. Beyond these roles, AhR functions extend to bile acids metabolism, since its overexpression has been shown to attenuate cholestatic liver injury by modulating bile acid pools and mitigating the associated liver damage [42]. These metabolic adjustments directly affect the local availability of endogenous AhR ligands, such as kynurenine, and may therefore plausibly modulate the activation threshold and functional polarization of hepatic MCs in regions exposed to gut-derived metabolites.

### 4.3. Regeneration, Proliferation, and Fibrosis

AhR’s influence extends to cellular dynamics, particularly in the context of tissue repair and pathology. It is expressed in liver progenitor cells, where it influences the expression of genes related to cell growth, metabolism, and tumorigenesis [43]. In liver regeneration models, AhR deficiency leads to increased proliferation and expansion of undifferentiated stem cells, suggesting that AhR acts as a brake on excessive cell growth, helping the fine-tune liver regrowth after injury [44]. AhR also modulates key signaling pathways such as Wnt/β-catenin and Hippo-YAP, which are central to liver cell proliferation and regeneration [44].

AhR plays a protective, anti-fibrotic role in the liver by preventing the activation of HSCs, the main fibrosis-inducing cell type. The loss of AhR activates HSCs, starting or aggravating liver fibrosis, while the activation of AhR with non-toxic ligands (e.g., ITE and YH439) inhibits HSC activation and reduces fibrosis in animal models [41,45,46]. Mechanistically, AhR disrupts the interaction of SMAD3 with β-catenin, blocking the expression of pro-fibrotic genes [45]. Additionally, AhR activation can induce ferroptosis (a form of cell death) specifically in HSCs, further limiting fibrogenesis without harming the hepatocytes [46]. Collectively, these mechanisms illustrate how AhR integrates signals governing cell fate, growth, and fibrogenesis, thereby coordinating appropriate tissue repair responses. Since MC-derived mediators, including tryptase, histamine, and IL-13, can activate or amplify fibrogenic pathways (Section 5.2), the AhR-dependent inhibition of HSC activation should be interpreted within the broader context of intercellular crosstalk. The net fibrogenic outcome may therefore depend on the balance between direct AhR signaling in HSCs and its modulatory effects on MCs, which are influenced by ligand availability and the activation state.

**Table 2:** Summary of liver cell types expressing AhR and their functional roles. AhR is broadly expressed in the liver, with high levels in hepatocytes and quiescent HSCs, and it is present in various immune and progenitor cell populations. Its expression is functionally important for liver metabolism, immune regulation, regeneration, and fibrosis.

Liver Cell Type	Evidence for AhR Expression	Main Functions of AhR in That Cell Type	Citations
Hepatocytes	High mRNA/protein expression; hepatocyte-specific AhR knockout abolishes Cyp1a1/1a2/1b1 induction by dioxin and shifts multiple metabolic pathways	Xenobiotic metabolism (CYP1 family), regulation of lipid and glucose metabolism, mitophagy via BNIP3, contributes to steatosis or protection from alcohol/APAP-induced injury depending on the ligand/context	[34,37,39,47]
Hepatic stellate cells (HSCs)	High AhR mRNA and protein expression; ~4 fold higher than that in hepatocytes; functional CYP1 induction; HSC-specific AhR deletion	Maintains quiescence, prevents activation and fibrogenesis, modulates TGF β/Smad3–β catenin signaling, and can induce HSC ferroptosis to resolve fibrosis	[29,33,45,47]
Kupffer cells/macrophages	AhR is expressed in liver macrophages; myeloid-specific AhR or AhRr knockouts modify liver damage and fibrosis; AhR activation alters Kupffer cell numbers and inflammatory profiles	Tunes inflammatory responses (IL 6, TNF, and IL 10), macrophage activation, fibrosis and steatosis during diet-induced obesity, toxic and autoimmune hepatitis	[12,41,47,48]
Liver sinusoidal endothelial cells (LSECs)	AhR expression has been detected proteomically and functionally; endothelial-specific AhR deletion disrupts ductus venosus closure	Vascular remodeling, embryonic closure of ductus venosus; likely roles in inflammatory and fibrotic signaling in the sinusoidal niche	[33,37,41,47]
Cholangiocytes	Proteomic analysis shows that the AhR protein is present at lower levels	Not well defined; may participate in biliary and inflammatory signaling, but its functions remain unclear	[33,47]
Other immune cells in the liver (T cells, B cells, ILCs, NKT cells, etc.)	AhR is broadly expressed in many immune subsets; liver scRNA-seq shows AhR-responsive gene changes in T, B, NK/NKT, and myeloid cells after TCDD	Balances pro- and anti-inflammatory responses and is involved in IL-17/IL-22 production, the Treg/Th17 balance, chemotaxis and cytokine profiles in autoimmune and inflammatory liver disease	[11,47,48]

### 4.4. Immunomodulatory Functions of AhR in Hepatic Immune Cells

Single-cell RNA sequencing reveals AhR expression in various liver immune cells, including B cells, T cells (CD4+, CD8+), natural killer T (NKT) cells, and memory T cells, where it modulates inflammatory responses [47,48]. Liver-resident macrophages, known as Kupffer cells, are the major orchestrators of hepatic inflammation and are likely influenced by AhR signaling; but direct evidence for high constitutive AhR expression specifically in Kupffer cells in vivo is less well defined than for HSCs and hepatocytes [45,48].

AhR is expressed in several innate immune populations, including NK cells and type 1 innate lymphoid cells (ILC1s), both of which are implicated in liver homeostasis and injury. In chronic liver damage, AhR activation enhances NK cell accumulation and cytotoxic function in the liver, whereas conditional AhR knockout reduces liver damage and fibrosis [48]. In acute injury models, excessive kynurenine production and AhR activation exacerbate inflammation and fibrogenesis. Pharmacological blockade of AhR attenuates liver injury, suggesting a context-dependent role for this axis [1]. These apparently divergent findings likely reflect differences in the timing, intensity, and cellular targets of AhR activation, with transient signaling contributing to immune regulation, whereas sustained or excessive kynurenine accumulation may amplify inflammatory and fibrogenic circuits. Moreover, AhR influences adaptive immunity by promoting regulatory T cells (Tregs) and suppressing effector T cell responses. This promotes tolerance in inflammatory environments and contributes to immune evasion in tumors and chronic infections [30,31,42,49]. In the liver, this immunosuppressive effect may blunt anti-viral or anti-tumor responses, favoring the persistence of hepatotropic viruses or tumor development.

In patients with autoimmune hepatitis (AIH), the imbalance between Tregs and T helper type-17 (Th17) has been linked to dysfunction of the AhR pathway resulting from aberrant inhibition or non-canonical activation. These alterations impair Treg- and Th17-mediated upregulation of CD39, an ectoenzyme critical for immunoregulation. Modulating the excessive inhibition or aberrant non-canonical activation of AhR may therefore represent a novel therapeutic strategy to control inflammation while restoring immune balance in AIH [50] (Table 2). Although these findings are primarily derived from studies of T cells, they raise the possibility that AhR-dependent pathways in other hepatic immune populations, including MCs, could contribute to immune dysregulation in AIH. However, direct evidence for MC-intrinsic AhR involvement in AIH remains limited.

Collectively, these findings show that AhR signaling shapes hepatic–immune interactions in a context-dependent manner. Although these pathways are not MC-intrinsic, they define the inflammatory milieu in which hepatic MCs reside and respond to local and gut-derived cues. Accordingly, gut-derived AhR ligands may influence MC activation both directly and indirectly through their effects on surrounding immune cell populations. Within this framework, MCs may act as potential integrators of microbial and immune signals along the AhR-dependent gut–liver axis—a concept that is developed in the following section.

## 5. Mast Cells and AhR Signaling in the Liver

Building on the hepatic context outlined in Section 4, this section focuses specifically on MCs and their AhR-dependent responses, and how they may intersect with gut-derived signals and liver pathology.

### 5.1. Overview of MC Biology and Modulation by AhR Signaling

MCs are long-lived innate immune cells that are present in most vascularized tissues, arising from embryonic yolk sac progenitors that can persist into adulthood and from later bone marrow-derived CD34+ progenitors, homing to mature tissues [51,52]. MCs express multiple receptors (e.g., high-affinity receptor for IgE (FcεRI) and low-affinity receptor for IgG (FcγRII), Toll like receptors (TLRs), the G protein-coupled receptor MGPRS, and cytokine/neuropeptide/adhesion receptors), allowing them to sense diverse stimuli and release preformed (histamine, tryptase, and chymase) and de novo generated mediators (lipid mediators, cytokines, chemokines, and growth factors), explaining their broad roles in physiology and disease [53,54]. Accordingly, MCs drive allergic reactions via FcεRI-mediated activation [55], contribute to host defenses against microbes, parasites and venoms by recruiting and activating other immune cells and modifying vascular responses [56,57], participate in vascular regulation and tissue repair through growth factors/cytokines [56,58], and communicate with neurons influencing pain/itch [59]; overall, they function as adaptable sentinels/modulators shaping initiation, amplification, the resolution of immune responses, and crosstalk with adaptive immunity [60].

Evidence that MCs express functional AhR has relatively recently been obtained: a 2012 study of the rat basophilic leukemia cell line RBL-2H3 showed that AhR agonists (e.g., kynurenine) induce Cyp1a1 expression and alter calcium flux, cytokine (e.g., IL-6) levels, and degranulation [61]. While RBL 2H3 is a basophilic, histamine-releasing cell line that shares features with both MCs and basophils without fully representing either cell type, AhR expression was detected in human and murine MCs in the same year. In these cells, FICZ elicited context-dependent effects—enhancing acute allergen-triggered degranulation but suppressing it after repetitive stimulation—and promoted a late pro-inflammatory cytokine program (IL-6, IL-17, and IL-13) [62].

In vivo, AhR-null mice show MC deficiency with impaired calcium signaling, mitochondrial damage, ROS accumulation and apoptosis, reduced c-Kit/STAT expression, and poor IgE/antigen responsiveness; conversely, AhR activation in wild-type mice potentiates IgE-mediated responses and worsens anaphylaxis [63]. However, the outcomes vary by the ligand, exposure time, and endpoint. In an RBL-2H3 model, kynurenine/kynurenic acid upregulate Cyp1a1 and violacein (a resveratrol-like molecule) downregulates it with distinct kinetics; functionally, kynurenic acid can enhance IgE-dependent degranulation, whereas kynurenine and resveratrol can suppress degranulation/cytokine production, while IL-6 regulation also differs across ligands [61]. In primary bone marrow-derived MCs, kynurenine (not kynurenic or quinolinic acid) potentiates IgE-mediated activation (degranulation and leukotriene and cytokine production) and augments cutaneous anaphylaxis [64].

In the gut, MCs constitutively express AhR, and ligand/duration-dependent AhR activation tunes degranulation and the IL-6/TNF-α/IL-17 output, influencing local inflammation and homeostasis [61,62,65], and, through the release of mediators, can shape dendritic cell and T-cell activation/trafficking, indirectly affecting T-cell polarization and the tolerance–inflammation balance [62,66,67,68,69].

### 5.2. MCs and AhR-Mediated Immune Regulation in Liver Diseases

**Figure 1:**
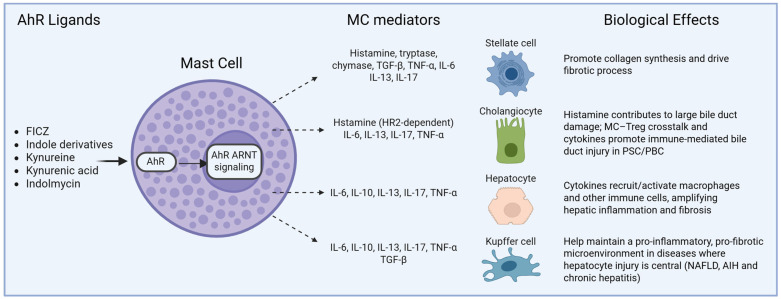
Proposed model of AhR signaling in MCs and its putative effect on liver cell types. Endogenous and microbe-derived ligands, including FICZ, indole derivatives, kynurenine, kynurenic acid, and indolmycin, activate the AhR, which translocates to the nucleus and forms a heterodimer with ARNT. This signaling pathway can modulate MC responses, leading to the release of pre-stored mediators (histamine and tryptase), pro-inflammatory cytokines (IL-6, IL-13, and TNF-α), and immunomodulatory mediators (IL-10 and TGF-β). Although these mediators have been independently implicated in liver pathophysiology, their regulation by AhR signaling in MCs and their effects on hepatic cell types are presented here as a conceptual framework, and they potentially contribute to context-dependent immune and inflammatory regulation in liver diseases (see text for details). Created with BioRender.com.

MCs are widely distributed throughout epithelial tissues, and they represent resident immune cells in the liver that are particularly located in periportal and peribiliary areas. In the liver, MCs interact with hepatocytes, cholangiocytes, HSCs, and Kupffer cells, contributing to modulating inflammation and fibrogenesis in several conditions. Multiple studies in both rodent and human models support a contributory role for MCs in the progression of various chronic liver diseases. The number of MCs significantly increases in the liver across a wide spectrum of pathologies, including nonalcoholic fatty liver disease (NAFLD), alcoholic liver disease (ALD), primary biliary cholangitis (PBC), primary sclerosing cholangitis (PSC), as well as chronic hepatitis and fibrosis progression [70,71,72,73,74]. The pathogenic contribution of MCs is mediated by the release of potent mediators: elevated levels of substances like histamine, tryptase, chymase, and MC-derived cytokines (e.g., TGF-β, TNF-α, and interleukins) are detected in diseased livers and are directly implicated in promoting local inflammation, fibrosis, and tissue remodeling [70,72,74]. The density of MCs within the portal tracts and fibrous septa shows a strong correlation with the degree of liver fibrosis and overall disease severity in both humans and animals [73,75,76]. Mechanistically, MCs contribute to tissue injury by releasing these pro-fibrotic and pro-inflammatory mediators, which in turn activate HSCs, promoting collagen synthesis and driving the fibrotic process [72,74]. The causal role of MCs in hepatic fibrosis is further supported by interventional studies, as the experimental depletion or inhibition of MCs or their mediators in animal models successfully reduces liver fibrosis and injury [77,78,79], positioning them as highly relevant cellular targets for therapeutic development. A conceptual model summarizing hypothesized effects of AhR-dependent MC activation on different hepatic cell types is shown in Figure 1.

As mentioned above, one of the pathogenic mechanisms of AIH is the reduced activity of CD39 on Tregs. CD39 degrades extracellular nucleotides, limiting purinergic inflammation and supporting Treg-mediated immune tolerance. Impaired CD39 expression or activity in AIH contributes to defective Treg suppressive functions and sustained hepatic inflammation. Recent studies have demonstrated that altered AhR signaling contributes to this defect in AIH; specifically, a preferential interaction of AhR with estrogen receptor-α, rather than with regulatory elements of the CD39 gene, leads to the downregulation of CD39 expression in Tregs [50,80]. Supporting the relevance of dysregulated AhR signaling in AIH, a recent study by Gao et al. demonstrated that blocking the aryl hydrocarbon receptor repressor (AhRR) on Th17 cells from AIH patients restores a physiological response to AhR signaling in B cells, highlighting AhRR as a potential therapeutic target [81].

In vitro studies further link AhR signaling to PBC. She et al. demonstrated that the environmental toxin tetrachlorodibenzo-p-dioxin activates AhR on T cells, leading to increased secretion of IFN-γ, IL-17, and other cytokines [82]. This supports a potential association between AhR signaling and PBC-related immune dysregulation in T cells. Whether similar AhR-dependent mechanisms operate in hepatic MCs in PBC remains speculative and requires direct investigation.

Collectively, these findings place AhR at the center of a regulatory network linking CD39^+^ Tregs and Th17 cells, integrating environmental sensing with the balance between immune tolerance and inflammation in liver diseases. Despite extensive evidence on the role of AhR in Tregs and Th17 cells, the role of AhR in MCs within the liver remains poorly characterized. Investigating the AhR-mediated regulation of MCs could provide critical insights into its contributions to inflammation, fibrosis, and immune tolerance in hepatic diseases, potentially revealing novel therapeutic targets.

## 6. Microbiota-Derived AhR Signaling in the Gut–Liver Axis: Implications for MC Activity and Liver Inflammation

In recent years, accumulating evidence has linked AhR activity to liver inflammation through its activation by gut microbial metabolism and environmental factors. Dietary-derived tryptophan is metabolized by the gut microbiota into indole-3-acetic acid (IAA) and tryptamine, which translocate to the liver and act as AhR ligands [83]. Studies in animal models have shown that the modulation of the gut microbiota composition and/or IAA levels can influence hepatic steatosis and fibrosis progression [84,85]. Further studies have highlighted the crucial role of microbiota-derived tryptophan metabolites in modulating immune responses in AIH via AhR activation in T cells. Li et al. demonstrated that levels of indole-3-carboxaldehyde (ICA), a microbial tryptophan catabolite, are significantly reduced in AIH patients [86]. ICA supplement restricted T-cell activation by promoting AhR nuclear translocation and the induction of PI3K interacting protein 1 (Pik3ip1), which inhibits the PI3K/Akt/mTOR pathway. This mechanism reduced hepatic inflammation and tissue damage in mouse models of T-cell-mediated hepatitis and was dependent on AhR expression in T cells. Moreover, the administration of *Lactobacillus reuteri* increased ICA levels and protected against liver injury, supporting microbiota modulation and AhR agonism as therapeutic strategies. Complementary findings reported by Pandey et al. showed that a deficiency of the epigenetic regulator Tet2 induces liver microbiome dysbiosis and enrichment of AhR ligand-producing pathobionts, such as *Lactobacillus reuteri*, which release indole-3-aldehyde (I3A) [80]. I3A promoted the differentiation of IFN-γ-producing CD8^+^ T cells and the development of AIH-like pathology in an AhR-dependent manner. The blockade of either IFN-γ or AhR reversed disease progression, underscoring the central role of the microbiota–AhR axis in AIH pathogenesis.

In humans, reduced levels of tryptophan metabolites have been detected in the stools of patients with metabolic syndrome, together with increased secretion of the incretin hormone GLP-1, which plays roles in glucose homeostasis and liver function [87].

Butyrate, a short-chain fatty acid (SCFA) produced by microbial fermentation, exerts anti-inflammatory effects on the gut by reducing TNF-α and IL-1β production. After absorption in the colon, butyrate enters the portal circulation and reaches the liver, where it acts as a histone deacetylase (HDAC) inhibitor. In human hepatoma cells (HepG2-C3) and primary human hepatocytes, butyrate increases AhR mRNA and protein levels in a dose-dependent manner and upregulates AhR target genes, including CYP1A1, CYP1B1, and AhRR. These effects are attenuated by AhR antagonists and AhR siRNAs, indicating AhR-dependent signaling [88]. In models of the gut–liver axis, butyrate also reduces systemic and hepatic inflammatory mediators and improves metabolic homeostasis [42,89]. In addition to hepatocytes, SCFAs, particularly butyrate, have emerged as important modulators of MC function. Experimental studies indicate that butyrate reduces MC reactivity by attenuating IgE-mediated degranulation and limiting the release of histamine, lipid mediators, and pro-inflammatory cytokines [90,91]. These effects are largely attributed to HDAC inhibition and the epigenetic reprogramming of MC activation pathways. Accumulating evidence suggests that SCFA-induced epigenetic changes may intersect with AhR-dependent signaling. By modifying chromatin accessibility and transcriptional responsiveness, butyrate may influence AhR target gene expression or alter MC sensitivity to endogenous AhR ligands derived from the diet or microbiota. Within the gut–liver axis, where MCs are exposed to microbial metabolites and AhR ligands, this SCFA–AhR interplay could represent a critical checkpoint controlling MC-driven inflammation. A dysbiosis-associated reduction in butyrate production increases MC reactivity and contributes to chronic inflammatory liver diseases. However, direct mechanistic evidence linking butyrate-induced AhR activation to MC regulation remains limited, highlighting the need for targeted studies addressing AhR signaling in MCs within hepatic and gut microenvironments.

As discussed above, reduced AhR activity promotes HSC activation: AhR is highly expressed in HSCs, where it inhibits TGF-β-mediated activation and suppresses pro-fibrotic gene expression [45]. Animal models have shown that AhR loss promotes the epithelial-to-mesenchymal transition through the direct activation of TGF-β signaling [92]. Dysbiosis further contributes to liver injury, and AhR activity appears to be a key mediator of this process [66]. Regulation of the gut microbiota composition reduces alcohol-related liver injury and fibrosis in animal models, concomitant with increased levels of tryptophan metabolites [93]. Microbiota-derived indoles produced by commensal bacteria such as *Lactobacillus* and *Bacteroides* can activate AhR and modulate MC functions in experimental systems [86,94,95]. Alterations in the microbiota composition may therefore disrupt AhR ligand availability, potentially influencing MC activation states and contributing to chronic inflammation. However, the specific role of MC-intrinsic AhR signaling in PSC, PBC, or NASH remains to be defined.

There is increasing evidence that MCs play a dual role in the gut–liver axis, contributing both to liver injury and to the maintenance of gut epithelial integrity. Intestinal MCs protect the liver by limiting bacterial translocation and the entry of microbial metabolites into the portal circulation [71]. Conversely, excessive MC activation can promote histamine and cytokine release into the portal circulation, driving hepatic inflammation [70,71].

Interactions between MCs and Treg also contribute to immune-mediated bile duct injury, promoting inflammatory cholestatic diseases such as PSC and PBC [77,96,97].

### AhR-Targeting Interventions Modulate MC Activity and Liver Fibrosis

Probiotic (e.g., *Lactiplantibacillus plantarum*) administration increases the abundance of AhR ligand-producing bacteria, resulting in reduced hepatic inflammation in models of liver injury [93]. This intervention restores AhR activity and reduces fibrosis, highlighting the therapeutic potential of modulating the gut microbiota–AhR axis [93,98]. Tranilast, also known as [N-3, 4-dimethoxy cinnamoyl]-anthranilic acid, is an analogue of a tryptophan metabolite, considered an anti-allergic drug for its ability to inhibit MC activation and degranulation, and an agonist of AhR [99]. Tranilast has been used clinically as a MC stabilizer for the treatment of allergic conjunctivitis [100] and skin keloids [101], and it has been reported to protect against Schistosoma mansoni-induced liver fibrosis [102]. Some studies have also examined the potential of Tranilast in the treatment of hepatic disorders. In a rat model of acute liver injury induced by thioacetamide injection, the treatment of the animals with Tranilast reduced the levels of circulating IL-6 and IL-13 and improved hepatic encephalopathy [103]. In 2008, Uno et al. investigated the effects of Tranilast on nonalcoholic steatohepatitis using obese diabetic rats. In this study, Tranilast, which was administered for eight weeks at a dose of 420 mg/kg/day, inhibited the progression of hepatic fibrosis and suppressed HSC activation. Moreover, the therapy reduced the expression of TGF-β and its target molecules (such as α1 procollagen and plasminogen activator-1), decreasing liver inflammation, TNF-α expression, and Kupffer cell recruitment [104]. Notably, the genes involved in β-oxidation (peroxisome proliferator-activated receptor α and carnitine O-palmitoyltransferase-1) were upregulated [104]. These findings highlighted the potential of Tranilast as a therapeutic agent for human liver diseases.

To date, only one AhR-targeting drug has been approved for clinical use, i.e., Tapinarof. Tapinarof binds to and activates AhR, and its efficacy has been validated in psoriasis and atopic dermatitis, which represent the main models of skin inflammatory diseases [105,106]. Other drugs with AhR-modulating activity exist, although they do not primarily target AhR, but the possibility of positively modulating AhR opens new perspectives in the treatment of other inflammatory disorders in humans, including hepatitis.

## 7. Conclusions

**Figure 2:**
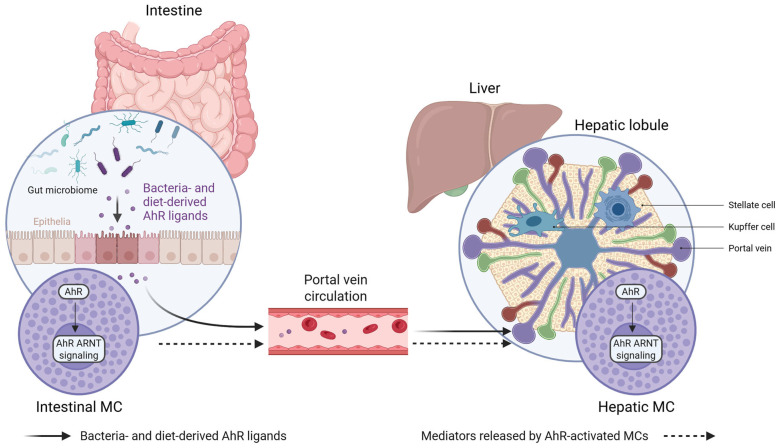
Schematic representation of gut microbiota-derived AhR signaling along the gut–liver axis. The gut microbiota and dietary components generate endogenous ligands for the aryl hydrocarbon receptor (AhR) within the intestinal lumen. These metabolites may activate AhR–ARNT signaling in intestinal epithelial cells and mast cells (MCs), resulting in the release of immunomodulatory mediators. Through the portal circulation, microbial metabolites and MC-derived factors reach the liver, where they may interact with hepatic MCs and other non-parenchymal cells, including Kupffer cells and HSCs. This conceptual model highlights a possible link between gut-derived AhR signaling and hepatic immune regulation. Created with BioRender.com.

The AhR–kynurenine axis plays a multifaceted and context-dependent role in liver inflammation. Under physiological conditions, the controlled activation of AhR by endogenous ligands contributes to immune tolerance and the resolution of inflammatory responses. However, excessive, sustained, or compartment-specific activation—particularly during acute injury or chronic disease—may instead promote inflammatory amplification or fibrogenesis. These divergent outcomes likely depend on the ligand concentration, duration of signaling, disease stage, and the specific cellular targets involved, including immune cell subsets, HSC, and MCs. Within this dynamic framework, the gut microbiota emerges as a central modulator of hepatic immune homeostasis by regulating the availability of AhR ligands along the gut–liver axis, as represented in Figure 2.

Importantly, recent experimental and pharmacological studies demonstrate that AhR is not only a mechanistic node but also a druggable target in liver disease. Microbiota-based interventions, such as probiotic administration, can restore AhR signaling and attenuate liver inflammation and fibrosis, highlighting the therapeutic potential of manipulating microbial AhR ligand availability. In parallel, pharmacological AhR agonists with MC-stabilizing properties, such as Tranilast, have shown efficacy in multiple experimental models of liver injury, steatohepatitis, and fibrosis by dampening MC activation, suppressing pro-fibrotic pathways, and modulating hepatic immune cell recruitment. The recent clinical approval of Tapinarof further supports the feasibility of AhR-targeted therapies and underscores the translational relevance of this pathway beyond the skin.

Despite these advances, several critical questions remain unresolved. As highlighted throughout this review, most MC–AhR interactions relevant to liver disease remain inferred from indirect or extrahepatic evidence, and their liver-specific relevance requires direct experimental validation. A deeper understanding of cell-specific AhR signaling in MCs is still lacking, particularly in chronic liver diseases where MCs accumulate and actively shape inflammatory and fibrotic responses. Whether MC-specific AhR transcriptional programs or AhR-dependent MC mediators could serve as diagnostic or prognostic biomarkers warrants further investigation. Moreover, ligand selectivity represents a major challenge, as distinct endogenous, microbial, and xenobiotic AhR ligands can trigger qualitatively different, and sometimes opposing, biological outcomes.

From a translational perspective, future research should focus on whether the selective and context-dependent modulation of AhR, rather than global activation or inhibition, can be exploited to restrain pathogenic MC responses while preserving protective immune functions. Strategies based on microbiota modulation, dietary interventions, or cell-type-restricted AhR targeting may offer novel and safer avenues to fine-tune hepatic immune homeostasis. Ultimately, elucidating the precise contribution of the MC–AhR axis within the gut–liver network will be essential for translating these insights into effective therapeutic strategies for chronic inflammatory and fibrotic liver diseases.

## Figures and Tables

**Table 1:** Summary of the main endogenous, microbial, dietary, and pharmacological AhR ligands, their context-dependent immunomodulatory effects, and their primary sites of origin. This table also reports the predominant routes through which each ligand is delivered to the liver. The immunomodulatory effect (pro- or anti-inflammatory) often depends on the ligand type, dose, cellular/tissue context, and presence of other immune cues.

Ligand (Origin)	Pro-/Anti-Inflammatory Effect	Primary Site → Delivery to Liver	Key Notes/Mechanism	Citations
TCDD (environmental pollutant)	Both (context-dependent)	Systemic; delivered to liver via the bloodstream	Can induce immunosuppression (Treg) or persistent inflammation, depending on the dose and context	[3,15,16]
FICZ (endogenous, tryptophan metabolite)	Both (context-dependent)	Produced in the skin/gut; reaches the liver via the bloodstream	Promotes Treg or Th17 differentiation; anti-inflammatory in the gut/skin, pro-inflammatory in some immune settings	[15,17,18,27]
Kynurenine (endogenous, tryptophan pathway)	Anti-inflammatory	Systemic; produced in many tissues incl. the liver	Promotes Treg function and immune tolerance, reduces inflammation in chronic inflammation and cancer	[19,20]
Indole-3-carbinol (I3C, dietary)	Anti-inflammatory	Gut lumen/epithelium; metabolites reach the liver via the blood	Suppresses pro-inflammatory cytokines, increases IL-10 levels, promotes Treg function	[22,29]
Indole derivatives (microbial/dietary, e.g., indole-3-acetic acid, IAA, indole-3-aldehyde, 3-IAld)	Mostly anti-inflammatory (context-dependent)	Produced in the gut by the microbiota; reaches the liver via the bloodstream	Act on intestinal immune cells and the epithelium; often support barrier integrity and Treg responses in gut-associated inflammation	[26]
Indirubin/indigo naturalis (microbial/dietary)	Anti-inflammatory	Gut and skin; metabolites can reach the liver via the bloodstream	Suppress pro-inflammatory mediators, increase Treg numbers, effective against colitis and skin inflammation	[21,22]
Resveratrol (dietary)	Anti-inflammatory	Absorbed in the gut; systemic, delivered to the liver via the blood	Induces Treg production, reduces inflammation, modulates the T-cell balance	[15,19]
Quercetin, bilirubin (dietary/endogenous)	Anti-inflammatory	Vascular system; delivered to the liver via the bloodstream	Vascular protection, reduce inflammation	[13]
Benzo[a]pyrene (environmental pollutant)	Pro-inflammatory	Lung/airways; systemic distribution, reaches the liver via the blood	Increases pro-inflammatory cytokine levels, promotes lung inflammation	[13,23,28]
Tapinarof (pharmacological)	Anti-inflammatory	Primarily skin; systemic absorption is possible	Reduces skin inflammation, improves barrier function	[21]
NPD-0414-2/24 (pharmacological)	Anti-inflammatory	Gut mucosa; may reach the liver via the bloodstream	Induces IL-22 expression, reduces IFN-γ expression, protects the gut	[24]
AGT-5 (pharmacological)	Anti-inflammatory	Systemic; likely delivered to the liver via the bloodstream	Promotes Treg production, reduces Th17/Th1, safe in animal models	[26]
2AI (pharmacological)	Anti-inflammatory	CNS (microglia); systemic exposure	Reduces the expression of pro-inflammatory genes and NO levels in microglia	[25]

## Data Availability

No new data were created or analyzed in this study.

## Abbreviations

The following abbreviations are used in this manuscript:

AhR	Aryl hydrocarbon receptor
AhRR	Aryl hydrocarbon receptor repressor
AIH	Autoimmune hepatitis
ARNT	Aryl hydrocarbon receptor nuclear translocator
bHLH-PAS	Basic helix–loop–helix/Per-ARNT-Sim
CYP1A1	Cytochrome P450 family 1 subfamily A member 1
CYP1B1	Cytochrome P450 family 1 subfamily B member 1
DRE	Dioxin response element
GLP-1	Glucagon-like peptide 1
HDAC	Histone deacetylase
HSC	Hepatic stellate cell
IAA	Indole-3-acetic acid
ICA	Indole-3-carboxaldehyde
IDO	Indoleamine 2,3-dioxygenase
IFN-γ	Interferon gamma
IgE	Immunoglobulin E
IL	Interleukin
ILC1	Type 1 innate lymphoid cell
I3A	Indole-3-aldehyde
MC	Mast cell
MeSH	Medical Subject Headings
NASH	Nonalcoholic steatohepatitis
NF-κB	Nuclear factor kappa-light-chain-enhancer of activated B cells
NK	Natural killer
NKT	Natural killer T
PAS	Per-ARNT-Sim
PBC	Primary biliary cholangitis
PI3K	Phosphoinositide 3-kinase
Pik3ip1	PI3K interacting protein 1
PSC	Primary sclerosing cholangitis
SCFA	Short-chain fatty acid
siRNA	Small interfering RNA
TCDD	2,3,7,8-Tetrachlorodibenzo-p-dioxin
TDO	Tryptophan 2,3-dioxygenase
TGF-β	Transforming growth factor beta
Th17	T helper 17
Treg	Regulatory T cell
TNF-α	Tumor necrosis factor alpha
